# *L*. *plantarum* WCFS1 enhances Treg frequencies by activating DCs even in absence of sampling of bacteria in the Peyer Patches

**DOI:** 10.1038/s41598-018-20243-1

**Published:** 2018-01-29

**Authors:** Miriam Bermudez-Brito, Theo Borghuis, Catherine Daniel, Bruno Pot, Bart J. de Haan, Marijke M. Faas, Paul de Vos

**Affiliations:** 1 Top Institute Food and Nutrition, Wageningen, The Netherlands; 2 Dept. Pathology and Medical Biology, University Medical Center Groningen and University of Groningen, Groningen, The Netherlands; 3 CNRS, INSERM U1019, CHU Lille, Institut Pasteur de Lille, UMR8204 - CIIL - Center for Infection and Immunity of Lille, University of Lille, Lille, France; 4 Research Group of Industrial Microbiology and Food Biotechnology, Faculty of Sciences and Bioengineering Sciences, Vrije Universiteit Brussel, Brussels, Belgium

## Abstract

Probiotics such as *L*. *plantarum* WCFS1 can modulate immune responses in healthy subjects but how this occurs is still largely unknown. Immune-sampling in the Peyer Patches has been suggested to be one of the mechanisms. Here we studied the systemic and intestinal immune effects in combination with a trafficking study through the intestine of a well-established immunomodulating probiotic, i.e. *L*. *plantarum* WCFS1. We demonstrate that not more than 2–3 bacteria were sampled and in many animals not any bacterium could be found in the PP. Despite this, *L*. *plantarum* was associated with a strong increase in infiltration of regulatory CD103^+^ DCs and generation of regulatory T cells in the spleen. Also, a reduced splenic T helper cell cytokine response was observed after *ex vivo* restimulation. *L*. *plantarum* enhanced Treg cells and attenuated the T helper 2 response in healthy mice. We demonstrate that, in healthy mice, immune sampling is a rare phenomenon and not required for immunomodulation. Also in absence of any sampling immune activation was found illustrating that host-microbe interaction on the Peyer Patches was enough to induce immunomodulation of DCs and T-cells.

## Introduction

Probiotics are live microorganisms which, when administered in adequate amounts, confer health benefits on the host^1^, such as enhanced clearance of pathogens, promoting intestinal epithelial survival and enhancing barrier function^2^. Of particular interest are the effects of probiotics on the gut immune system. How the probiotic bacteria enhance immunity and how they interact with the gut immune system remains elusive^3,4^.

It is hypothesized that probiotics may modulate the immune system through two different pathways: (i) probiotics might be sampled by M cells in the Peyer’s patches (PPs) follicle-associated epithelium and modulate macrophages and dendritic cells (DCs) beneath the epithelium^5^ or (ii) specific intestinal DCs in the mucosal lamina propria or PP sense intraluminal probiotics by pattern-recognition receptors (PRRs) on their dendrites^6,7^. This contact with DCs, via either of both pathways, may regulate the maturation of antigen-presenting cells (APCs), and subsequently influence interactions with other effectors of the immune system, polarizing the subsequent antigen-specific T cell response towards Th1, Th2, Th17 or T regulatory cells^8^.

A better understanding of the mechanistic basis of host-bacteria interactions that regulate intestinal immune processes is crucial for the development of effective probiotic strategies. However, *in vivo* studies on this are rare^9–12^ as most studies addressing mechanisms of action of probiotics are performed *in vitro* and mainly use non-intestinal cells^13^ such as peripheral blood mononuclear cells (PBMCs)^14^, spleen cells^15^, and peritoneal macrophages^16^. These cells do not necessarily produce the same responses as intestinal cells upon exposure to probiotics.

The current study was designed to evaluate which sampling pathway(s) is responsible for immune effects, i.e. sampling of probiotic bacteria in the PP or sensing of probiotics by the lamina propria DCs, without sampling. To this end, we investigated the systemic and intestinal immune effect in combination with a trafficking study through the intestine of a well-established probiotic strain, *L*. *plantarum* WCFS1, labeled with the luciferase from *Pyrophorus plagiophthalamus* emitting in the red spectra. We studied the effect of these bacteria on the systemic adaptive immune system after 5 days of oral administration, i.e. the period required to develop a T cell response in mice^17,18^.

## Materials and Methods

### Ethics statement

This study was carried out in accordance with the recommendations of FELASA guidelines and the ethical committee for animal experiments from the University of Groningen (DEC-RUG). The protocol was approved by the ethical committee for animal experiments from the University of Groningen (DEC-RUG).

#### Bacterial Strain and Growth Conditions

The *L*. *plantarum* was made bioluminescent as described before^19^. Shortly, the *L*. *plantarum* codon-optimized *cbrluc* gene under the control of *pldh* were cloned into pNZ8148 as *BglI*I-*Xba*I fragment. The resulting construct subsequently was introduced into *L*. *plantarum* NCIMB8826 by electrotransformation as described elsewhere^20^ and named *L*. *plantarum*-CBRluc (Lp-CBRluc). *L*. *plantarum* NCIMB8826 containing the empty vector pNZ8148 (named Lp-pNZ8148), served as controls in all of the experiments. Strain stability was tested as described previously^19^.

*L*. *plantarum* was grown at 37 °C in De Man Rogosa and Sharpe (MRS) medium (Difco, Becton Dickinson). Chloramphenicol (Sigma-Aldrich, St. Quentin Fallavier, France) was added to culture media for bacterial selection, at a final concentration of 10 g/ml.

*L*. *plantarum* WCFS1^21^ without the construct was cultured at 37 °C in MRS broth until stationary growth. Subsequently, the cultures were diluted 1:1000 in fresh medium and cultured for a second night. The optical density at 600 nm was measured and the number of colony forming units (CFU) was calculated based on standard growth curves. For all cultured bacterial strains, an OD_600_-value of 1 corresponds to 1–2 × 10^9^ CFU/mL, which was confirmed by plating serial dilutions on MRS or M17 agar plates (data not shown)^22^. The mice daily received either sterile MRS or 1–2 × 10^8^ CFU bacteria in 200 µL MRS via intra-gastric gavage.

#### Animals

Wild-type male Balb/c mice were purchased from Harlan (Harlan, Horst, The Netherlands). The animals were fed standard chow and water *ad libitum*. All animal experiments were performed after receiving approval of the institutional Animal Care Committee of the Groningen University.

*L*. *plantarum* WCFS1 in MRS broth only (carrier), were administered by intragastric gavage of a 200 µL volume once daily. The carrier and the bacterial strains were administered for five consecutive days. At day six, the mice were sacrificed, after which the spleen and PPs were removed for further analysis.

#### *In vivo* bioluminescence imaging

Bioluminescence imaging was performed using a multimodal IVIS Lumina XR (Caliper, PerkinElmer) as described before^19^, which consists of a cooled charge-coupled-device camera mounted on a light-tight specimen chamber. Prior to bioluminescent imaging, mice were anesthetized with 2% isoflurane. d-Luciferin potassium salt (Caliper, PerkinElmer) at 30 mg/ml was then administered to animals inoculated with CBRluc-expressing strains by intragastric inoculation (200 μl/mouse). Anesthetised mice (induction with isoflurane) were placed into the camera chamber of the IVIS, where a controlled flow of 1.5% isoflurane in air was administered through a nose cone via a gas anesthesia system. A grayscale reference image under low illumination was taken as an overlay prior to quantification of emitted photons over 1 s to 5 min, depending on signal intensity and using the software Living Image (Caliper, PerkinElmer). For anatomical localization, a pseudocolor image representing light intensity (blue, least intense, to red, most intense) was generated using the Living Image software and superimposed over the grayscale reference image. For each individual mouse, there was only one ROI corresponding to the mouse digestive tract, and this ROI was determined manually. Bioluminescence was quantified using the Living Image software (given as p/s).

#### Cell isolation and restimulation

Following sacrifice of the mice, spleens or PPs were removed. Single cell suspensions were made by mechanical disruption of the tissue between two glass slides in 1 mL of ice cold RPMI containing 10% heat inactivated fetal calf serum (FCS). Subsequently a cell strainer was used to remove remaining clumps. The cells were washed, counted, and used for staining.

Part of the cells of the spleen were stimulated, the remaining cells were left unstimulated. An amount of 7 × 10^6^ spleen cells were stimulated in RPMI with 10% FCS containing 40 nM Phorbol 12-myristate 13-acetate (PMA) (Sigma Aldrich) and 2 nM calcium ionophore (Ca^2+^) (Sigma Aldrich). Monensin (3 µM) (Sigma Aldrich) was added to allow cytokine accumulation in the cellular cytoplasm. Cells were stimulated for four hours at 37 °C, after which they were washed twice in ice cold PBS containing 2% heat inactivated FCS (FACS buffer), and used for staining.

Dead cells were removed from the cell suspensions. For this, 1 mL of cell suspension was loaded on 1 mL of 1-step Polymorphs (Accurate Chemical and Scientific Corporation, Westbury, NY) with a density of 1.068 ± 0.001 g/ml, and centrifuged for 30 minutes at 300 × *g* at 4 °C. The interface was washed twice in ice cold FACS buffer and used for staining. After density gradient centrifugation, more than 90% of the cells was vital, which was confirmed by propidium iodide staining.

#### Cell staining

T-cell staining was performed on non-stimulated, non-enriched splenic cell suspensions. Staining for intracellular cytokines were performed on PMA/Ca^2+^ stimulated splenic and cell suspensions. The T cell cocktail contained monoclonal antibodies against CD3, CD4, CD8, CD25, CD69, Foxp3, or appropriate isotype controls. The effector T cell cocktail contained monoclonal antibodies against CD3, CD4, CD8, IFN-γ, IL5, IL10, IL17A, IL22, or appropriate isotype controls (Table 1).

**Table 1:** Antibodies used for flow cytometry.

Specificity	CloneName	Fluorochrome	Dilution	Supplier
CD3	17A2	Pacific Blue	200x	BioLegend
CD4	RM4–5	PerCP	200x	BioLegend
CD8	53–6.7	Alexa700	50x	BioLegend
CD25	3C7	APC	100x	BioLegend
CD69	H1.2F3	PE	200x	BioLegend
FoxP3	FJK-16S	FITC	100x	eBioscience
Rat IgG2b	N/A	APC	100x	BioLegend
Hamster IgG	N/A	PE	200x	BioLegend
Rat IgG2a	N/A	FITC	100x	eBioscience
IFN	XMG1.2	APC	100x	BioLegend
IL5	TRFK5	PE	25x	BioLegend
IL10	JES5–16E3	PE	25x	BioLegend
IL17A	TC11–18H10.1	APC	25x	BioLegend
IL22	140301	PE	25x	R&D Systems
Rat IgG1	N/A	APC	100x	BioLegend
Rat IgG1	N/A	PE	25x	BioLegend
Rat IgG2a	N/A	PE	25x	BioLegend
Rat IgG2b	N/A	PE	25x	BioLegend
CD11c	N418	APC	25x	BD Biosciences
MHC II	2G9	Biotin + streptavidin PerCP	150x	BD Biosciences
CD19	6D5	PE-Cy7	100x	BioLegend
CD80	16–10A1	PE	50x	BioLegend
CD86	PO3	Alexa700	50x	BioLegend
CD103	2E7	Pacific Blue	25x	BioLegend
Hamster IgG	N/A	PE	50x	BioLegend
Rat IgG2b	N/A	Alexa700	50x	BioLegend
Hamster IgG	N/A	Pacific Blue	25x	BioLegend

The DC cocktail applied on the PPs cell suspension contained CD11c, MHC II, CD19, CD80, CD86, CD103, or appropriate isotype controls (Table 1).

In short, 1 × 10^6^ cells were incubated in FACS buffer containing 10% normal mouse serum for 30 minutes to prevent non-specific antibody staining. Subsequently, the cells were incubated with a cocktail of primary antibodies for 30 minutes, in the dark, after which the cells were washed in ice cold FACS buffer twice. Tubes stained for T cells were subsequently fixed in ice cold 1 × FACS Lysing solution (BD Biosciences) for 30 minutes in the dark, washed twice in ice cold FACS buffer and resuspended in the same buffer until analysis. Also, the tubes for intracellular cytokine staining were fixed in ice cold 1 × FACS Lysing solution, for 30 minutes in the dark and washed twice in ice cold 1 × permeabilisation buffer (eBioscience). Subsequently the cells were incubated with the intracellular antibody cocktails containing 2% normal rat serum in permeabilisation buffer for 30 minutes in the dark. Then cells were washed twice in ice cold permeabilisation buffer and resuspended in FACS buffer until analysis. Tubes for DC-staining were washed twice in ice cold FACS buffer, after which cells were incubated with the secondary step for 30 minutes in the dark. Subsequently, cells were fixed in ice cold 1 × FACS Lysing solution for 30 minutes in the dark followed by washing twice in ice cold FACS buffer and resuspended in FACS buffer until analysis.

#### Flow cytometry

**Figure 1:**
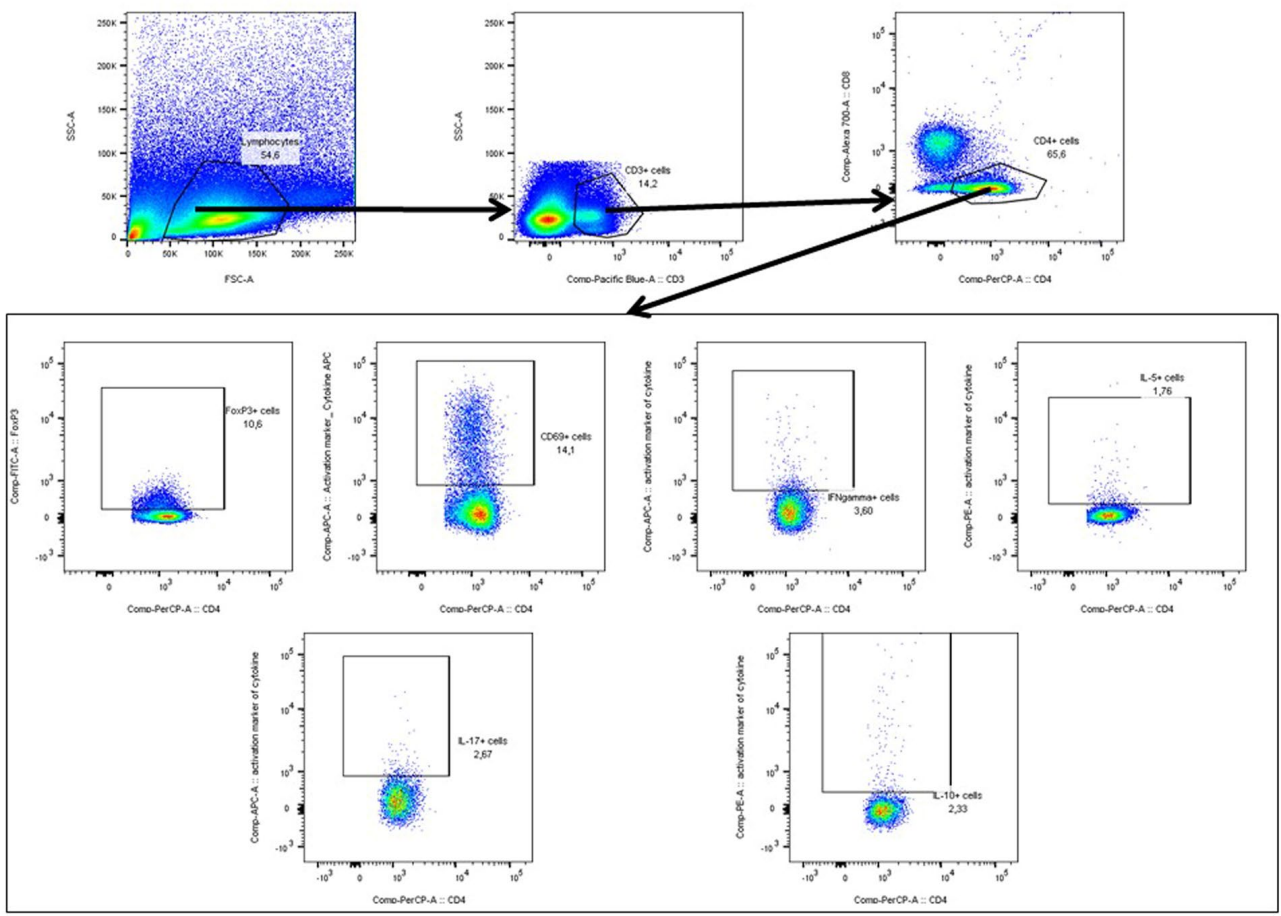
Lymphocytes were gated in the forward side scatter plot and the frequency of CD3^+^ T cells was determined. Within the T cell population, the frequency of CD8^+^ T cells and CD4^+^ T cells was determined. Within both the CD4 and CD8 T cell population the isotype controls for CD69, CD25, or the cytokines, were used to set the gate to 99% negative cells. This gate was then copied to the sample stained for CD69, CD25, or cytokines and the frequency of positive cells was determined. Further, within the CD4 T cell population the FoxP3 isotype control was used to set the gate to 99% negative cells. This gate was copied to the sample stained for FoxP3 and the frequency of positive cells was determined.

**Figure 2:**
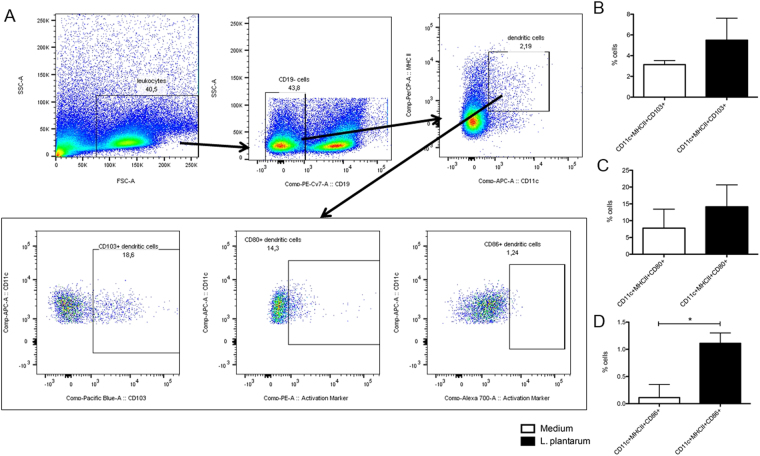
L. plantarum does induce dendritic cell activation in the Peyer Patches even without sampling of the bacteria. Frequency of dendritic cell subsets in the spleen (N = 6) following oral treatment with medium (white bars), or L. plantarum WCFS1 (black bars). Dendritic cells were gated based on the expression of CD11c and MHC II (**A**). DCs were gated in the forward side scatter plot, based on size and granularity, and the frequency of MHC II^+^ CD11c^+^ cells was determined. CD19^+^ B-cells were excluded from analysis. Within the DC populations CD80, CD86, or CD103 isotype controls were used to set the gate to 99% negative cells. This gate was copied to the sample stained for CD80, CD86, and CD103 and the frequency of positive cells was determined (**A**). Dendritic cell frequencies are depicted as the frequency of CD103^+^ cells within the CD11c^+^ MHC II^+^ cell compartment in the spleen (**B**). Activated dendritic cells are depicted as the frequency of CD80^+^ cells within the CD11c^+^ MHC II^+^ cell compartment in the spleen (**C**), or the frequency of CD86^+^ cells within the CD11c^+^ MHC II^+^ cell compartment in the spleen (**D**). Results are depicted as the mean ± standard error of the mean (SEM). Statistical significance was calculated using the Students t-test. *Represents P-values < 0.05.

During flow cytometry, at least 5 × 10^5^ cells were analyzed. Flow Cytometry was performed using the LSR II Flow Cytometer system (BD Pharmingen), using FACS Diva software. Analysis was performed using the FlowJo 7.6.2 software. Lymphocytes were gated in the forward side scatter plot and the frequency of CD3^+^ T cells was determined. Within the T cell population, the frequency of CD8^+^ T cells and CD4^+^ T cells was determined. Within both the CD4 and CD8 T cell population the isotype controls for CD69, CD25, or the cytokines, were used to set the gate to 99% negative cells. This gate was then copied to the sample stained for CD69, CD25, or cytokines and the frequency of positive cells was determined. Further, within the CD4 T cell population the FoxP3 isotype control was used to set the gate to 99% negative cells. This gate was copied to the sample stained for FoxP3 and the frequency of positive cells was determined (Fig. 1). DCs were gated in the forward side scatter plot, based on size and granularity, and the frequency of MHC II^+^ CD11c^+^ cells was determined. CD19^+^ B-cells were excluded from analysis. Within the DC populations CD80, CD86, or CD103 isotype controls were used to set the gate to 99% negative cells. This gate was copied to the sample stained for CD80, CD86, and CD103 and the frequency of positive cells was determined (Fig. 2A).

#### Definition of immune cell populations

In this study, different immune cell populations are defined based on the expression of specific cell surface markers. Splenic and mesenteric regulatory T cells (Treg) are defined based on the expression of CD25 and the transcription factor FoxP3. For this, FoxP3^+^ cells were gated within the CD4 T cell population and the expression of CD25 was confirmed. All CD4^+^FoxP3^+^ cells consistently demonstrated CD25 expression. Results are depicted as the frequency of CD25^+^FoxP3^+^ cells within the total CD4 T cell population (CD4^+^CD3^+^ cells). Activated T cells are defined as the frequency of CD25^+^FoxP3^−^ or CD69^+^ cells within the CD4^+^CD3^+^ (T helper cells; Th) or CD8^+^CD3^+^ (cytotoxic T cells; CTLs) population. Effector cell populations are defined as the frequency of IFN-γ^+^IL5^−^, IFN-γ^−^IL5^+^, IL10^+^, and IL17^+^IL22^+^ cells within the CD4^+^CD3^+^ or CD8^+^CD3^+^ cell population. DCs were defined as CD11c^+^ MHC II^+^ cells. Regulatory, intestinal DCs are depicted as the frequency of CD103^+^ cells within the CD11c^+^ MHC II^+^ population. Also, the frequency of activated DCs was determined and depicted as the frequency of CD80^+^ or CD86^+^ cells within the CD11c^+^ MHC II^+^ cell population.

#### Immunohistochemistry staining

To stain bioluminescent *L*. *plantarum* in PPs, PPs were frozen in precooled iso-propane, sectioned at 5 mm, and processed for immuno-histochemical staining. As primary antibody, we applied Anti-Firefly Luciferase antibody ab181640 (Abcam) in a 1:250 dilution. As second antibody, we applied FITC-conjugated rabbit anti-goat antibody (1:100, Dako) and as third biotinylated swine anti-rabbit antibody (1:100, Dako). Tissue slides of 2 Å~ were then washed for 10 min with PBS and incubated with DAPI solution (30 nM) for 5 min to visualize nuclei of the epithelial cells. After washing with PBS, samples were mounted with mounting medium. Slices were studied with a SP8 Leica confocal microscope (Leica Microsystems, Son, the Netherlands).

#### Statistics

All data are expressed as the mean ± standard error of the mean (SEM). Normal distribution of the data-sets was confirmed by the Kolmogorov-Smirnov test. The two-sided Students t-test was used to determine changes in immune cell populations after probiotic treatment *in vivo*. P-values < 0.05 were considered statistically significant.

## Results

### Bioluminescent *L*. *plantarum* trafficking through the GI tract of healthy mice

**Figure 3:**
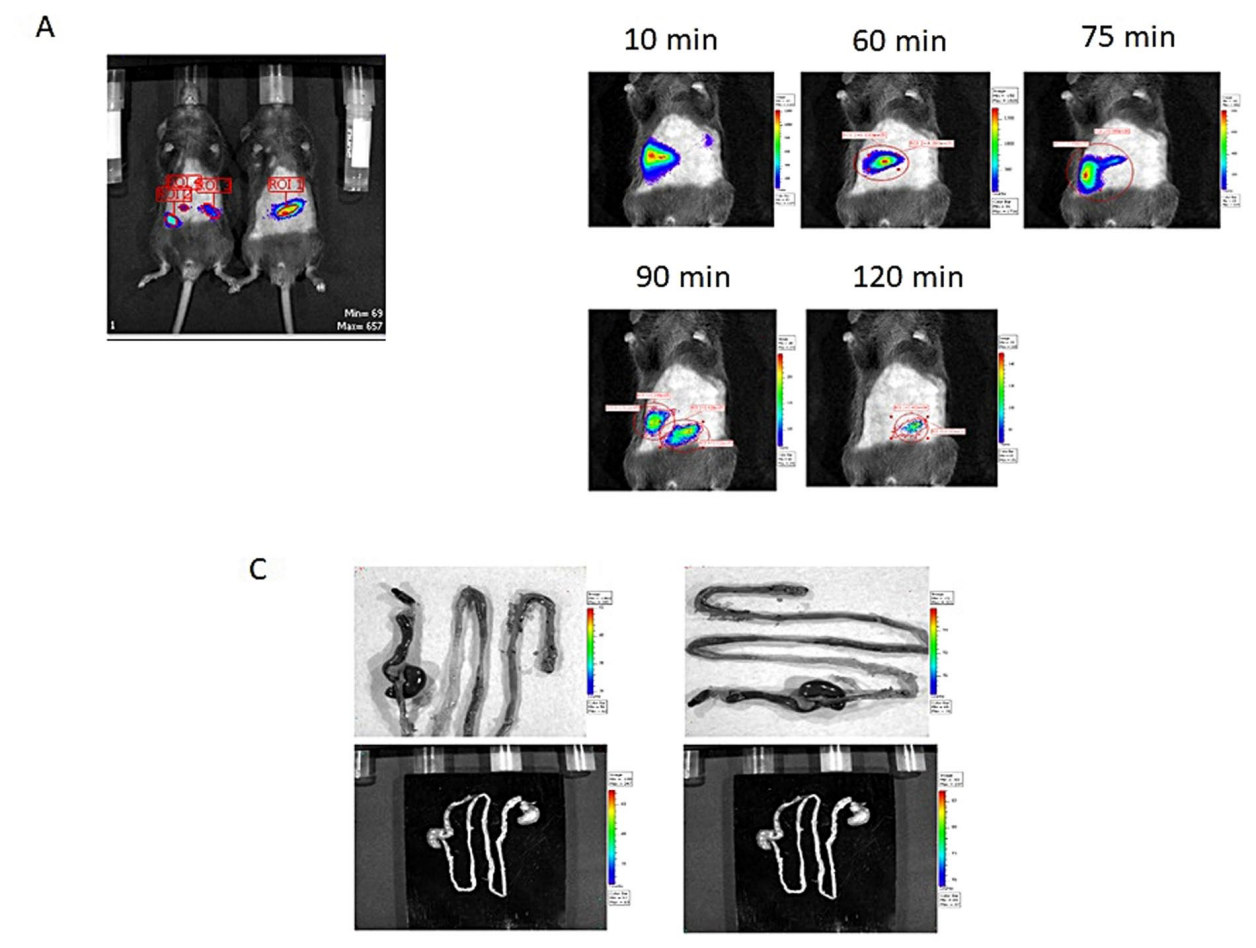
Bioluminescent L plantarum trafficking through the GI tract of healthy mice. Monitoring of intestinal transit of *L*. *plantarum* by bioluminescence imaging in whole animals. *L*. *plantarum*-CBRluc (1–2 × 10^8^ CFU) was inoculated intragastrically into mice, and the bioluminescent signal was measured transcutaneously in whole animals at different time points postfeeding. (**A**) The intensity of the transcutaneous photon emission is represented as a pseudocolor image. Two representative mice are shown. (**B**) Transit of *L*. *plantarum* in the digestive tract of mice. Mice were fed once with 1–2 × 10^8^ CFU of *L*. *plantarum*-CBRluc. One representative image of one mouse is shown after 10, 45, 60, 75, 90, and 120 min. d-Luciferin was given intra-gastrically 1 h before administration of bacteria, and the bioluminescence signal was measured 3 h after inoculation of the substrate. (**C**) The digestive tract of the mouse was then dissected after sacrifice, and the bioluminescent signal was quantified on intact organs. A representative mouse is shown. No signal was detected.

**Figure 4:**
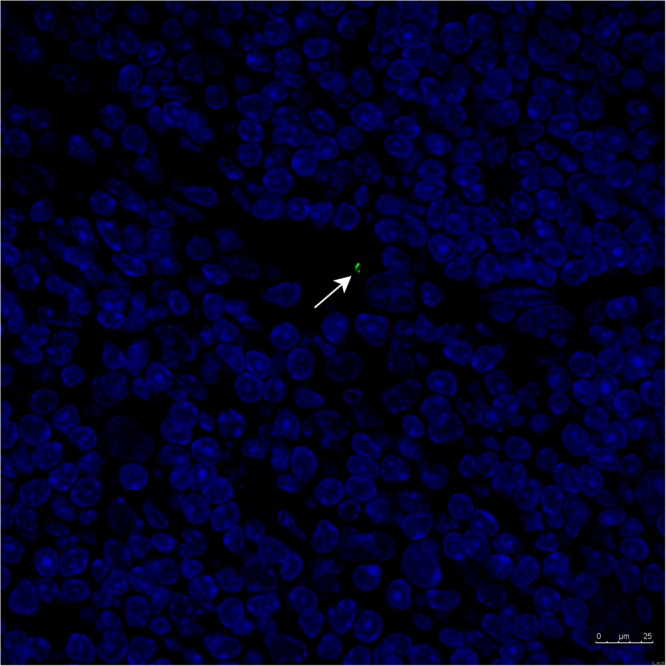
Uptake of the bacteria in the PPs. *L*. *plantarum*-CBRluc (1–2 × 10^8^ CFU) was inoculated intra-gastrically into mice. After 5 days, mice were sacrificed and PPs were excised. All PPs were completely sliced and stained. PPs were frozen in precooled iso-propane, sectioned at 5 mm, and processed for immuno-histochemical staining. As primary antibody, we applied Anti-Firefly Luciferase antibody ab181640 (Abcam) in a 1:250 dilution. As second antibody, we applied FITC-conjugated rabbit anti-goat antibody (1:100, Dako) and as third biotinylated swine anti-rabbit antibody (1:100, Dako). Representative image showing that in only two of the eight animals we observed remnants or intact bacteria in the PPs.

Intra-gastric gavage of 1–2 × 10^8^ CFU bacteria was performed and followed by IVIS. *L*. *plantarum* had a transit time of two hours (Fig. 3A,B). After this period, we did not detect any bioluminescent signal. After the fifth day, the intestine was removed and studied again by IVIS. In the isolated intestines, without interference of the skin, we observed no accumulation of the bacteria in immune sampling area, around PPs areas (Fig. 3C). Next, we excised the PPs to study uptake of the bacteria in this immune sampling organ. In only two of the eight animals we observed remnants or intact bacteria in the PPs (Fig. 4). All PPs were completely sliced and stained but we only found 2–4 bacteria in the two animals and nothing in the remaining 6 animals.

### *L*. *plantarum* treatment affects the frequency of regulatory T cells and T-cell polarization in the spleen

To determine whether, despite the absence of true sampling of bacteria in the PP, immunomodulation occurred, in a follow-up experiment we studied changes in T-cell frequency in the spleen in mice following *L*. *plantarum* WCFS1 administration (doses of 1–2 × 10^8^ CFU).

**Figure 5:**
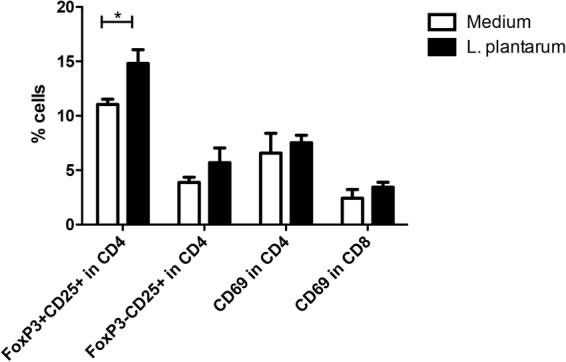
L. plantarum WCFS1 induced increased frequencies of regulatory T cells in the spleen while had no effect on the frequency of pro-inflammatory activated T cells. Regulatory T cell frequencies in the spleen (N = 6) following oral treatment with medium (white bars), or L. plantarum WCFS1 (black bars). Regulatory T cell frequencies are depicted as the frequency of CD25^+^FoxP3^+^ cells within the CD4 T cell compartment, CD69^+^ cells within the CD4 T cell compartment (C&D), and CD69^+^ cells within the CD8 T cell compartment. Results are depicted as the mean ± standard error of the mean (SEM). Statistical significance was calculated using the Students t- test. *Represents P-values < 0.05.

*L*. *plantarum* WCFS1 induced (n = 6 mice) increased frequencies of Treg cells in the spleen as compared to medium treated animals. *L*. *plantarum* WCFS1 had no effect on the frequency of pro-inflammatory activated T cells such as CD25^+^ CD69 + FoxP3^−^ and CD69^+^ cells in the CD4^+^ (T helper; Th) and CD69+ CD8^+^ CTL populations (Fig. 5).

In order to determine the consequences of *L*. *plantarum* WCFS1 administration on T cell responses, we performed another series of experiments. Cells from the spleen were restimulated with PMA/Ca^2+^ *ex vivo*, after which the IFN-γ, IL5, IL10, and IL17 response of CD4^+^ and CD8^+^ T cells was determined.

**Figure 6:**
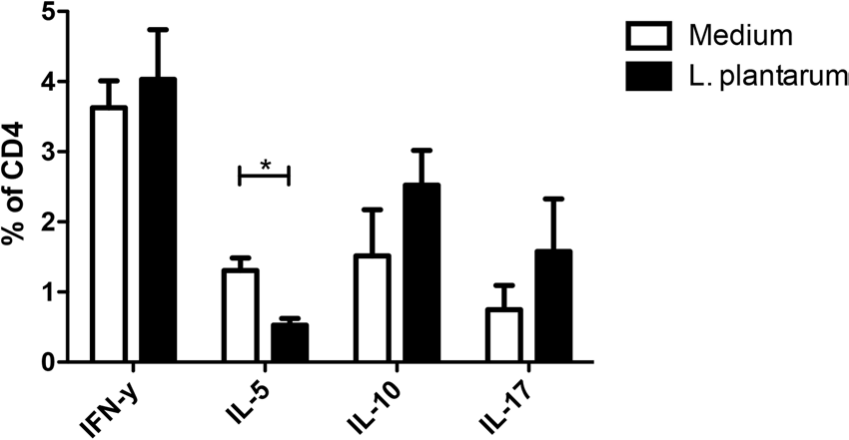
L. plantarum WCFS1 treatment had an attenuating effect on Th2 responses. Polarized CD4 T cell frequencies in the spleen (N = 6) following oral treatment with medium (white bars), or L. plantarum WCFS1 (black bars). Polarized CD4 T cell frequencies are depicted as the frequency of IFNγ^+^ cells within the CD4 T cell compartment, IL5^+^ cells within the CD4 T cell compartment, IL10^+^ cells within the CD4 T cell compartment, and IL17^+^ cells within the CD4 T cell compartment. Results are depicted as the mean ± standard error of the mean (SEM). Statistical significance was calculated using the Students t-test. *Represents P-values < 0.05.

**Figure 7:**
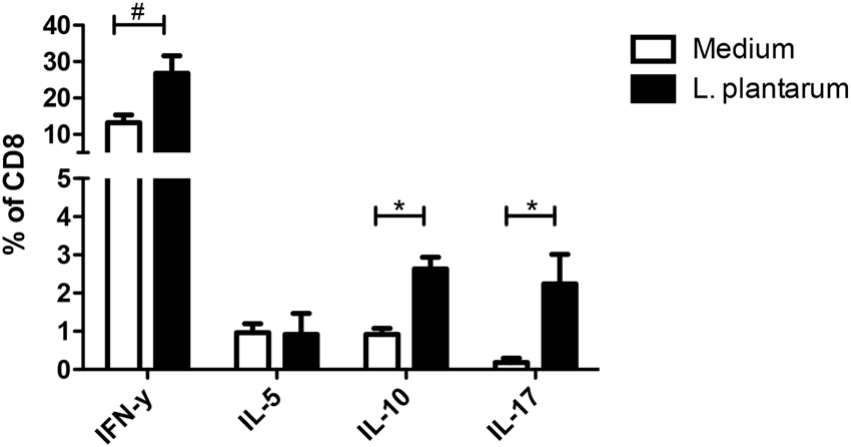
L. plantarum had a more pronounced effect on CD8+ T cells. A 2-fold increase in frequencies of IFN-γ producing cells was observed in the CD8+ T cells as well as an increase in IL10 producing CD8+ T cells. Polarized CD8 T cell frequencies in the spleen (N = 6) following oral treatment with medium (white bars), or L. plantarum WCFS1 (black bars). Polarized CD8 T cell frequencies are depicted as the frequency of IFNγ^+^ cells within the CD8 T cell compartment, IL5^+^ cells within the CD8 T cell compartment, IL10^+^ cells within the CD8 T cell compartment, and IL17^+^ cells within the CD8 T cell compartment. Results are depicted as the mean ± standard error of the mean (SEM). Statistical significance was calculated using the Students t-test. *Represents P-values < 0.05, **Represents P-values < 0.01.

The *L*. *plantarum* WCFS1 treatment had an attenuating effect on Th2 responses (IL5 producing CD4+ T cells), and demonstrated a trend towards increased IL10^+^ Th cell frequencies in the spleen (p < 0.1). Other polarized CD4 Th subsets were not affected (Fig. 6). *L*. *plantarum* had a more pronounced effect on CD8+ T cells. A 2-fold increase in frequencies of IFN-γ producing cells was observed in the CD8+ T cells as well as an increase in IL10 producing CD8+ T cells and a 6-fold increase in IL17-producing CD8+ T cells. *L*. *plantarum* had no effect on IL5-producing CD8+ T cells (Fig. 7).

### *L*. *plantarum* does induce dendritic cell activation in the Peyer Patches even without sampling of the bacteria

As immunomodulation was observed in the spleen, even in mice where no bacteria were found in the PPs, we questioned whether despite absence of immune sampling some immune upregulation had occurred in the PPs. To this end, we isolated PPs of mice treated for 5 days with *L*. *plantarum* and determined the frequency of CD11c+ MHC II+ DCs, CD103+ DCs, CD80+ DCs, or CD86+ DCs. These DCs are the first immune cells to respond to transcytosed antigens in the dome area, which is just below the follicular epithelium^23^. The percentage of CD103+ intestinal DCs was increased, while the percentage of CD103- intestinal DCs was decreased although did this not reach statistical significance (Fig. 2B). Although no effect of probiotic treatment was observed on % CD80+ DCs in the PP (Fig. 2C), the treatment with *L*. *plantarum* WCFS1 did increase the activation status of the DCs in the PPs as demonstrated by increased frequencies of CD86+ DCs (Fig. 2D).

## Discussion

Understanding the mechanistic basis for host-bacteria interactions that regulate intestinal immune processes is crucial for the development of novel therapeutic strategies with probiotics^3^. Although many studies demonstrate beneficial immune responses induced by probiotics, a key question that still needs to be addressed is how they interact with the gut immune system^23,24^. Current theories can be divided into those that propose an active immune sampling, mediated by M cells in the PPs, modulating macrophages and DCs beneath the epithelium^25^, or those mechanisms in which specific DCs in the lamina propria or PP take up probiotic antigens^6,7,26–28^. DCs have also been proposed to sample translocated bacteria that reach the lamina propria^29^. The current study was designed to evaluate whether sampling of probiotic bacteria in the PP is an actual part of the mechanism by which probiotics induce immune changes in the mucosa or that the second proposed mechanism, i.e. sensing by DCs without sampling, is responsible for the immune effects. Our data suggest that true sampling by PP M-cells of *L*. *plantarum* is a rare phenomenon but that DCs in PPs are activated, suggesting that sensing of DCs with PRRs^3^ without active sampling is responsible for immune effect observed in the mice.

Neutra *et al*.^30^ and Hamada *et al*.^31^ suggested that sampling of commensal bacteria is likely to occur through M-cells of the PPs and lymphoid follicles. Surprisingly, in their studies actual proof for active large-scale sampling was lacking. Our study demonstrates that sampling of the probiotic strain *L*. *plantarum* WCFS1, which is also a commensal, does occur in the PP, but is rather rare. Our observation that immune cell populations changed despite rare sampling in the PP corroborates the findings of Macpherson *et al*.^32,33^ demonstrating that only tiny proportions (approximately 0·0001%) of a challenge dose of intestinal bacteria actually reach DCs either in the villi or the PPs but are efficaciously inducing mucosal immune responses. As a proposed mechanism Macpherson *et al*. suggested that the DCs in the PPs and isolated lymphoid follicles promiscuously sample intestinal luminal commensals, whereas lamina propria DCs sample those bacteria located within the surface mucus layer^33^. However, in our experiments sensing via DCs in the PPs is by far the most probable mechanism of immune modulations by this strain as CD11c+ MHC II + DCs isolated from the PPs were increased as well the activation status as illustrated by enhanced CD86 expression. The CD103+ DCs that migrate to the mesenteric lymph nodes to generate T regs were decreased although this did not reach statistical significance. Macpherson *et al*. observed that commensals do not penetrate further than the mesenteric lymph nodes. The few bacteria that did penetrate were phagocytosed and eliminated to avoid inflammation and prevent destruction of their luminal habitat^32,33^.

To the best of our knowledge, this is the first report combining probiotics induced immune responses with a localization study in the intestine of a luciferase-expressing *Lactobacillus*, *L*. *plantarum* WCFS1. To do so we applied a recently developed engineered *L*. *plantarum* WCFS1 with reporter genes-based labelling system^19^. Although the development of such a system was far from easy^19,20^, it is a very instrumental system to study immune sampling, persistence and trafficking of the bacteria through the gastrointestinal tract^34^. This system has been helpful to demonstrate not only that immune sampling is a rare phenomenon but also that persistence of the probiotic is minimal, if not absent. Within 2 hours the strain can pass though the mice intestine and reach the cecum/colon. Similar kinetic results have been shown by Daniel *et al*.^19^ and others^35,36^. We also found that the cecum and colon were the predominant sites for persistence of *L*. *plantarum* in mice^19,35^. Interestingly, by using transcript profiles of *L*. *plantarum* WCFS1 residing in the ceca of germ-free mice, Marco *et al*.^37^ demonstrated that wild-type *L*. *plantarum* modifies its gene expression in ways designed to minimize the levels of D-alanylated lipoteichoic acids (LTA) present on the cell surface *in vivo*, and therefore limit its exposure to components of the host’s innate and/or adaptive immune system.

It is assumed that the majority of lactobacilli are passengers in the GI tract and that rarely more than 1% of the total number of bacteria colonize^38^. Despite low colonization rates lactobacilli are able to exert pronounced effects on the physiological and immunological systems of the host^39^. This also applies to *L*. *plantarum* WCFS1 where after 5 days of administration, despite absence of colonization, the effects of the probiotic were found to be profound, with many intestinal and systemic effects, including enhanced T reg numbers. Our data suggest that just contact of the luminal bacteria with the DCs is enough to induce these effects^40,41^.

Our data also demonstrate that *L*. *plantarum* induced immune effect remote from the mucosal to the systemic circulation. In the spleen, i.e. the site of measurement for systemic effects in mice, *L*. *plantarum* induced a strong increase in infiltration of regulatory CD103^+^DCs and increased the number of regulatory T cells^17^. These CD103^+^DCs are probably not only the DCs that migrated from the PP but also CD103^+^ DCs that patrol underneath and between enterocytes, while extending dendrites toward the lumen, likely using specific tight junction proteins to penetrate between the epithelium^28^. These intraepithelial CD103+ DCs sample bacterial antigens for presentation in the mesenteric lymph nodes and the spleen. In addition, the observed changes were accompanied by a reduced splenic Th cell cytokine response, suggesting the promotion of a regulatory systemic phenotype in addition to the observed enhanced Treg numbers.

The observation that *L*. *plantarum* enhances Treg cells and attenuates the Th2 responsiveness in the systemic circulation suggests that this strain might prevent or reduce the frequency of typical Th2-mediated disorders such as asthma or allergy^42^. *L*. *plantarum* induces high levels of IL-12 and IFNγ suppressing Th2 differentiation^42,43^. The present results corroborate our previous findings that *L*. *plantarum* WCFS1 consumption directly increased the frequency of T reg cells, while decreasing the responsiveness of Th2 cells and increasing the responsiveness of the CTL compartment^17^. This demonstrates that *L*. *plantarum* not only stimulates skewing towards immune regulation, but can also directly improve the responsiveness of cytotoxic T lymphocytes by leaving the Th1 compartment unaltered. Also, several reports have indirectly demonstrated improved CTL responsiveness following probiotic treatment by demonstrating improved immune responses towards viral infections *in vivo*^44–48^.

Overall, our results highlight the fact that in absence of any immune sampling as observed in the majority of mice sensing of probiotics and DC activation still occurred and was enough to induce immunomodulation. With this study, we demonstrate that *L*. *plantarum* WCFS1 induce Treg responses at the site of interaction^49^, but also profoundly affects the systemic immune system by skewing it to a more regulatory phenotype.
